# The Brassica genome

**DOI:** 10.3389/fpls.2013.00148

**Published:** 2013-05-30

**Authors:** Xiaowu Wang, Michael Freeling

**Affiliations:** ^1^The Institute of Vegetables and Flowers, Chinese Academy of Agricultural SciencesBeijing, China; ^2^Plant and Microbial Biology, University of CaliforniaBerkeley, CA, USA

## Brassica genome research topic

*Brassica* species include important crops and provide unique materials for the study of genome evolution. These crops include six important vegetables and oilseed crops, which have been classically described by “U's triangle'. The three diploid species *B. rapa* (A genome), *B. nigra* (B genome), and *B. oleracea* (C genome) have formed the amphidiploid species *B. juncea* (A and B genomes), *B. napus* (A and C genomes), and *B. carinata* (B and C genomes) by hybridization. Moreover, the three diploid species themselves are evolved from a paleohexaploid, that is old enough to allowing the species being evolved into diploid species and young enough to maintain significant synteny with its ancestors. These make the *Brassica* species of the uniqueness of polyploidy in botanical evolution.

Brassicas are closely related to the model plant *Arabidopsis thaliana*, one of the most extensively studied species in the world. Sequencing of the genome of *Brassica rapa* provided a great opportunity to bridge the rich knowledge obtained from Arabidopsis to be transferred to a cultivated species. Yet, tools and resources need to be established to accomplish the knowledge transfer. The release of the *B. rapa* var. Chiifu genome is not only of importance for genome evolution research, but also facilitates the gene discovery and breeding of the Brassica crops. Conversely, using the duplicated *Brassica* species as “deletion machines” to better understand the cis/trans-relationships of ENCODE-like features that are accumulating all over the arabidopsis genome is an additional, fundamental reason for continued study of the *Brassica* species.

This Research Topic—The *Brassica* Genome—gathers contributions that report establishment of novel tools and methods, comparative genomics, gene discovery, molecular marker development, and genetic dissection of important traits. We hope this modest compendium marks the beginning of a vibrant future for *Brassica* comparative genome biology, and points the way toward how the Brassica lineage of crucifers, with arabidopsis as the outgroup, can and will revolutionize studies of eukaryotic gene and genome regulation and the phenotypes they specify.

